# Comparison of Sales Income and Research and Development Costs for FDA-Approved Cancer Drugs Sold by Originator Drug Companies

**DOI:** 10.1001/jamanetworkopen.2018.6875

**Published:** 2019-01-04

**Authors:** Kiu Tay-Teo, André Ilbawi, Suzanne R. Hill

**Affiliations:** 1 World Health Organization, Geneva, Switzerland

## Abstract

**Importance:**

High costs and risks of research and development (R&D) have been used to justify the high prices of cancer drugs. However, what the return on R&D investment is, and by extension what a justifiable price might be, is unclear.

**Objective:**

To compare incomes from the sales of cancer drugs with the estimated R&D costs.

**Design, Setting, and Participants:**

This observational study used global pharmaceutical industry sales data to quantify the cumulative incomes generated from the sales of cancer drugs for companies that have held patents or marketing rights (originator companies). All cancer drugs approved by the US Food and Drug Administration from 1989 to 2017 were identified from the United States Food and Drug Administration’s website and literature. Itemized product sales data were extracted from the originator companies’ consolidated financial reports. For drugs with data missing in specific years, additional data was sought from other public sources, or where necessary, estimated values from known reported values. Drugs were excluded if there were missing data for half or more of the years since approval. Data analysis was conducted from May 2018 to October 2018.

**Main Outcomes and Measures:**

Sales data were expressed in 2017 US dollars with adjustments for inflation. Cumulative incomes from the sales of these drugs were compared against the R&D costs estimated in the literature, which had been adjusted for the costs of capital and trial failure (risk adjusted).

**Results:**

Of the 156 US Food and Drug Administration–approved cancer drugs identified, 99 drugs (63.5%) had data for more than half of the years since approval and were included in the analysis. There was a median of 10 years (range, 1-28 years) of sales data with 1040 data points, 79 (7.6%) of which were estimated. Compared with the total risk-adjusted R&D cost of $794 million (range, $2827-$219 million) per medicine estimated in the literature, by the end of 2017, the median cumulative sales income was $14.50 (range, $3.30-$55.10) per dollar invested for R&D. Median time to fully recover the maximum possible risk-adjusted cost of R&D ($2827 million) was 5 years (range, 2-10 years; n = 56). Cancer drugs continued to generate billion-dollar returns for the originator companies after the end-of-market exclusivity, particularly for biologics.

**Conclusions and Relevance:**

Cancer drugs, through high prices, have generated returns for the originator companies far in excess of possible R&D costs. Lowering prices of cancer drugs and facilitating greater competition are essential for improving patient access, health system’s financial sustainability, and future innovation.

## Key Points

**Question:**

How does income from the sales of cancer drugs compare with the costs of research and development?

**Findings:**

In this observational study of 99 cancer drugs approved by the FDA from 1989 to 2017, the median income return by the end of 2017 was found to be $14.50 (range, $3.30-$55.10) for every $1 research and development spending. Many drugs, particularly biologics, continued to generate high-sales incomes for the originator companies after expiry of patents and exclusive marketing rights.

**Meaning:**

Cancer drugs, through high prices, have generated incomes for the companies far in excess of research and development costs; lowering prices of cancer drugs and facilitating greater competition are essential for improving patient access, health system’s financial sustainability, and future innovation.

## Teaser

This observational study determines whether sale incomes and research and development (R&D) costs for US Food and Drug Administration (FDA)–approved cancer drugs sold by originator drug companies justify high prices for cancer drugs.

## Introduction

The costs of research and development (R&D) for drugs has been a recurrent point of discussion in the continuing debate about high prices of drugs. Most recently, the Trump administration’s *Blueprint to Lower Drug Prices and Reduce Out-of-Pocket Costs* argues that foreign countries “Are not paying an appropriate share of the necessary research and development to bring innovative drugs to the market,”^1^ implying a direct association between the costs of R&D and drug prices.

Indeed, high costs and high risks of R&D^2,3,4^ have been presented to justify high prices especially for cancer drugs. It has been argued that drug prices must account for all R&D costs, including the costs of failed products that were investigated but not marketed. It has also been argued that drug prices should cover the costs of capital, that is, the potential financial returns had the money been used in other areas of investment with equal risk to drug development (ie, opportunity cost). Taking these considerations into account, the R&D costs of developing an approved drug have been estimated between $100 to 150 million and $4 to 6 billion.^5^ However, the most commonly accepted estimates of R&D costs, including cancer drugs,^6^ are between $200 million and $2.9 billion, after adjustments for the probability of failure and opportunity costs.^3,4,6,7^

In addition to covering R&D costs, high drug prices are also justified on the basis that financial return needs to be sufficiently high to incentivize and finance the R&D of new drugs.^8^ The industry has claimed that 20% of its revenues were reinvested into R&D.^9^ However, previous studies have found that the financial returns of some cancer drugs (eg, imatinib and enzalutamide) were more than sufficient to cover past R&D expenses for the originator companies and generate generous profit to incentivize future R&D.^6,10,11^ A systematic analysis of the return on R&D investment for cancer drugs has not yet been conducted, to our knowledge, and can inform a discussion on what a justifiable price could be. To this end, we undertook this study to quantify the income generated from the sales (ie, sales income net of rebates and discounts but without accounting for expenses and taxes) of cancer drugs for the pharmaceutical companies that have held patents or marketing rights (ie, originator companies).

## Methods

### Study Design

This study was conducted to quantify the reported global incomes from the sales of individual drugs approved by the US Food and Drug Administration (FDA) between 1989 and 2017 for the treatment and prevention of hematological cancers, solid tumors, and related conditions such as neutropenia and hypercalcemia (cancer drugs).

### Data Sources

We identified all drugs approved by the FDA between 1989 and 2017 for any cancer-related indications from the FDA website^12^ and published literature.^13^ We then retrieved consolidated annual financial reports from websites of the originator companies of the identified drugs. Where companies’ annual reports were unavailable, we retrieved annual filing of financial statements (ie, forms 10-K or 20-F) from the database of US Securities and Exchange Commission^14^ or other websites (eg, http://getfilings.com). We extracted itemized annual sales data for each cancer drug from the year of FDA approval to 2017. Where there was a transfer of marketing rights between companies, or comarketing and copromotion agreements, sales data from all companies involved were sought from their respective annual reports for cross validation. Where required, we also sought data from journal articles and gray literature, identified through searches on web search engine (ie, Google) using nonspecific search terms: the brand and generic name, year, and name of the company. Only literature in English was included.

For missing sales data in specific years owing to nonreporting or nonavailability of documents, we first estimated the sales of a drug according to the growth rate documented in the annual report for that drug in the following year. For the years without any publicly available information based on the above search method, we applied exponential interpolation between 2 known values. For products that have past the period of market exclusivity and were known to have remained in the originator companies’ portfolios, we applied exponential decay to estimate missing data. If data were missing for more than half of the years since approval, we excluded the product from the analysis.

For R&D costs of cancer drugs, we used the estimates presented in a 2017 study that calculated the median risk-adjusted R&D cost as $794 million (range, $219-$2827 million).^6^ This range is comparable with estimates quoted by the pharmaceutical industry.^3,4,7^

### Statistical Analysis

Sales data reported in British Pound, Euro, Japanese Yen, and Swiss Franc were converted to US dollars based on published currency exchange rates in the corresponding individual years.^15^ The reported sales data were adjusted to 2017 value according to the annual world’s percentage inflation in consumer prices.^16^ This is conservative because the rates were lower than the rates used for calculating capital costs of R&D in most years during the observation period (at 7%^6^ and 10.5%^4^). We compared the cumulative income from the sales of the cancer drugs with the estimated median and range of risk-adjusted R&D costs in 2017 value. All analyses and data visualization were performed in Excel 2010 (Microsoft).

### Uncertainty and Assumptions

A subset of drugs have on-label noncancer indications, eg, rituximab has an approved indication for rheumatoid arthritis. Specific sales data were obtained for cancer indications through different brand names (eg, zaltrap [ziv-aflibercept] and eylea [aflibercept]) or when the sales data were presented separately for oncology indications (eg, rituximab). For simplicity, we did not make adjustments in the base-case analysis for data that could not be disaggregated to the cancer-specific proportion. Instead, the results were presented with notes about the drugs’ noncancer indications where relevant. Sensitivity analyses were performed by incorporating the costs associated with additional postapproval randomized clinical trials for possible extension of indications or dosage forms. The financial costs of 1 Phase I, Phase II, and Phase III trial in oncology have been estimated as $4.5 million, $11.2 million, and $22.1 million, respectively, in 2014^17^ (or $4.6 million, $11.5 million, and $22.8 million, respectively, in 2017 dollars).

In addition, sensitivity analyses were conducted by assuming twice the base-case R&D cost estimates (ie, median, $1588 million; range, $438 million-$5654 million) based on the observation that the mean cost of oncology drug pivotal trials was about 1.5 to 2 times higher than the costs of pivotal trials in other therapeutic areas (except cardiovascular).^18^ We also examined the mean income return on investment by including the R&D costs of 57 drugs excluded from the base-case analysis; in this sensitivity analysis, we assumed in the absence of robust data that these drugs would accrue, unrealistically, 0 revenue during the observation period.

## Results

### Data Characteristics

From 1989 to 2017, 156 cancer drugs were approved by the FDA. Of these drugs, 99 (63.5%) had sales data that were publicly reported in more than half of the years since approval and were therefore included in the analysis. The median number of years of observation since market authorization for the included drugs was 10 years (range, 1 year for acalabrutinib, durvalumab, and niraparib to 28 years for goserelin), resulting in a total of 1040 data points. Seventy-nine data points (7.6%) were interpolated or extrapolated from reported sales data. Total sales from the set of included drugs in 2017 ($106.9 billion) represented 80.4% of the estimated global incomes for cancer drugs ($133 billion).^19^

### Sales Incomes

In 2017, median sales incomes across all drugs was $435.2 million (range, $1.8 million for topotecan to $8.2 billion for lenalidomide) (eTable in the Supplement). The mean annual sales income since the year of approval ranged between $3 million for Acalabrutinib (approved in October 2017) and $5.9 billion for bevacizumab (approved in February 2004). Thirty-three drugs (33.3%) were found to have mean annual sales income of more than $1 billion.

**Figure 1.**
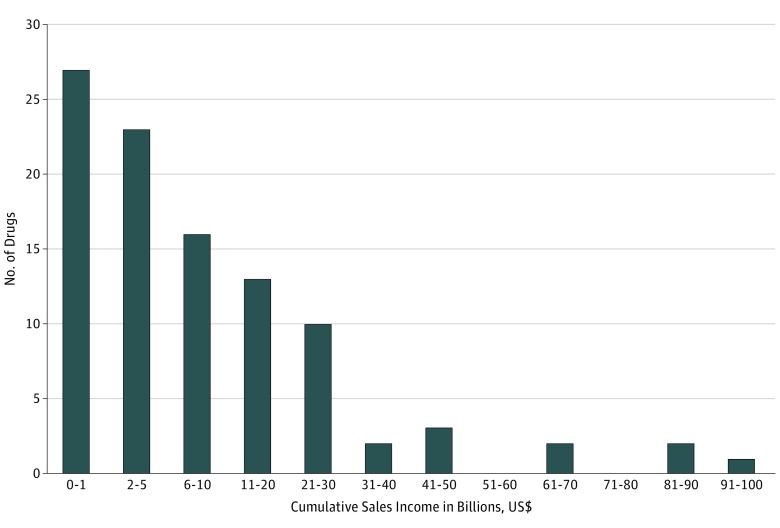
Distribution of Cancer Drugs by Cumulative Sales (1989-2017) Cumulative sales incomes were reported as US dollars in 2017 value.

**Figure 2.**
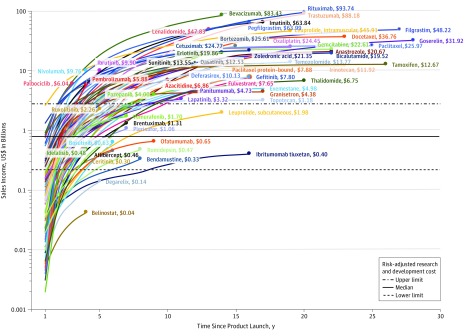
Cumulative Sales of Cancer Drugs in 2017 US Dollars, by Drug and Time Since Product Launch

Forty-nine cancer drugs (49.5%) had cumulative sales of more than $5.0 billion (Figure 1). At the end of 2017, 5 drugs had accrued sales incomes of more than $50 billion for the following originator companies: rituximab ($93.7 billion), trastuzumab ($88.2 billion), bevacizumab ($83.4 billion), pegfilgrastim ($64.0 billion), and imatinib ($63.8 billion) (Figure 2). Rituximab and trastuzumab had been available for about 20 years since approvals in 1997 and 1998 (by the FDA),^20^ while imatinib, pegfilgrastim, and bevacizumab received their first approval in 2001, 2002, and 2004,^20^ respectively. The reported incomes for bevacizumab included sales related to off-label use for macular degeneration of the eye, but income from this indication is likely to be low because of the smaller therapeutic dosage.

The cumulative sales incomes for 7 drugs appeared to be lower than the overall set of drugs (Figure 2)—ziv-aflibercept, belinostat, degarelix, ibritumomab tiuxetan, ofatumumab, and romidepsin. However, financial returns for these drugs might have been fully recovered. For example, the R&D costs of belinostat received substantial contributions from public sector funds.^21,22^ The income for ibritumomab tiuxetan is likely to be underestimated because sales income are those only from the United States and not the historical sales in Europe by (the now defunct) Schering AG. For ofatumumab, the sales trajectory might have been affected by transfer of marketing rights from GlaxoSmithKline to Novartis in 2014, possible strategic transitioning the drug for use in multiple sclerosis, and the introduction of alternative drug for chronic lymphocytic leukemia in 2014 (ie, ibrutinib).^23^

Many cancer drugs were found to have generated substantial financial returns for the originator companies after market exclusivity ended, particularly for biologics (Figure 2). For example, the principal patents taking filgrastim expired in August 2006 (Europe) and December 2013 (United States) following its launch in 1991.^24^ Although its income has declined following competition from biosimilar products, filgrastim continued to generate more than $500 million annually (range, $549-$1.2 billion) for the initial originator company after losing market exclusivity in 2014. The originator company of filgrastim also reported more than $4 billion in sales income annually from pegfilgrastim, whose principal patents expired in October 2015 (United States) and August 2017 (Europe).^24^ The reported annual sales incomes for anastrozole, bicalutamide, rituximab, trastuzumab, capecitabine, temozolomide, and thalidomide were also in the hundreds of millions of dollars for the originator companies following expiry of their principal patents.

### Returns on Investment

Based on a risk-adjusted R&D cost of $794 million ($2827 million-$219 million),^6^ by the end of 2017, $1 (risk-adjusted) invested for R&D of the 99 drugs had generated a median of $14.50 (range, $3.30-$55.10) in sales income for the originator companies. Assuming the upper threshold R&D cost of $2827 million, rituximab, trastuzumab, bevacizumab, pegfilgastim, and imatinib have brought in for the originator companies, $33.20, $31.20, $29.50, $22.60, and $22.60, respectively, for every risk-adjusted dollar that went into their R&D.

**Figure 3.**
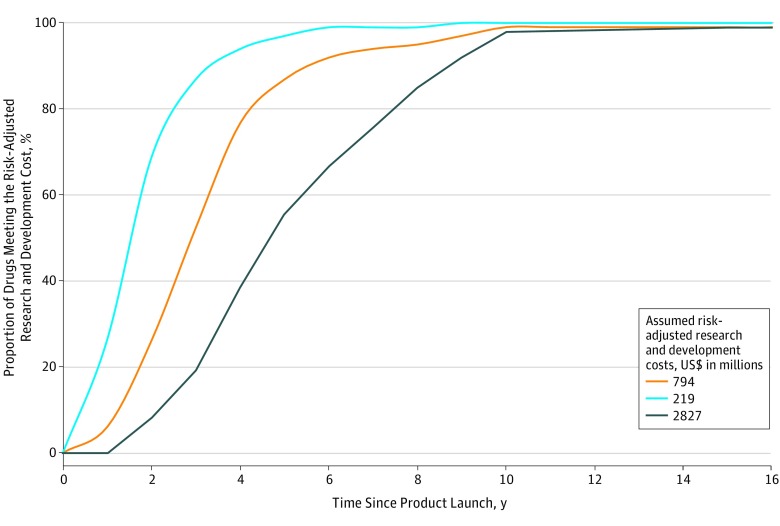
Threshold Analysis of Income Return, by Assumed Risk-Adjusted Research and Development Costs and Time Since Product Launch

By the end of 2017, the cumulative sales of 73 cancer drugs (73.7%) included in the analysis had fully recovered the median R&D costs of $794 million. If the assumed R&D costs were $2827 million and $219 million, 56 (56.6%) and 91 (91.9%) drugs, respectively, had at least generated incomes exceeding the R&D costs by the end of 2017 (Figure 3). The median time to generate sales income to fully cover risk-adjusted R&D cost of $794 million was 3 years (range, 2-5 years; n = 73). For the maximum estimated risk-adjusted cost of R&D ($2827million), the time to cost recovery was 5 years (range, 2-10 years; n = 56). A threshold analysis found that 99% of the 45 cancer drugs with sales data 10 years from their first year of launch had generated incomes sufficient to at least offset the risk-adjusted R&D costs irrespective of the assumed threshold values for R&D costs (Figure 3).

### Sensitivity Analyses

The median sales incomes were found to range from $11.40 to $13.80 for every dollar spent on R&D when the costs of up to 5 randomized clinical trials, for the extension of indications or development of additional dosage forms were incorporated (Table). The incomes remained at least 3 times higher than the estimated R&D costs for up to 5 postapproval trials, irrespective of whether the costs of phase I trials were incorporated. When the R&D costs for cancer drugs were assumed to be twice as high as the base-case estimates ($1588 million; range, $438 million-$5654 million), the median income return was $6.70 (range, $1.20-$27.10) per dollar invested for R&D. Finally, the median income, with accrual of R&D costs but with 0 sales incomes from the 57 drugs excluded from the base-case analysis, was found to be $8.80 (range, $1.70-$34.40) per R&D dollar.

**Table.** Sensitivity Analyses on Sales Return on Investment, by Number of Postapproval Trials for Extension of Indications or Dosage Forms

Clinical Trial Phases	No. of Postapproval Trials					
	0 (Base Case)	1	2	3	4	5
Additional R&D, No., US$ millions						
All phases	0	39	78	117	156	195
Phases II-III	0	34	69	103	137	172
Income return on investment, median (range), US$ millions						
All phases	14.5 (3.3-55.1)	13.8 (3.3-46.7)	13.1 (3.2-40.4)	12.5 (3.2-35.6)	11.9 (3.1-31.8)	11.4 (3.1-28.7)
Phases II-III	14.5 (3.3-55.1)	13.8 (3.3-47.5)	13.3 (3.2-41.7)	12.7 (3.2-37.2)	12.2 (3.1-33.5)	11.7 (3.1-30.5)

Abbreviation: R&D, research and development.

## Discussion

Despite the longstanding debates about the relationship between R&D costs and drug prices,^25,26,27^ to our knowledge, this is the first study that systematically quantified incomes from the sales of a comprehensive set of cancer drugs, over a long observation period, and compared with their estimated costs of R&D.

Our analysis has shown that incomes from the sales of cancer drugs greatly exceeded R&D costs. Based on an estimated median R&D cost of $794 million, we found that by the end of 2017 the median sales income across the 99 cancer drugs analyzed was $14.50 for every risk-adjusted R&D dollar invested. Thirty-three drugs had already qualified as “blockbuster drugs” according to the conventional definition of having an average annual sales income exceeding $1 billion. The financial returns from this set of drugs will continue to increase because many molecules have long remaining periods of market exclusivity (eg, ibrutinib, nivolumab, palbociclib, pembrolizumab). As observed in our analysis, many of these drugs would continue to accrue incomes in the hundreds of millions for the originator companies beyond the end of market exclusivity, particularly for biologics. This could be due to entry barriers for biosimilar products that restrict competition,^28^ and other factors that reinforce market dominance of the originator companies, such as contractual arrangements and clinicians’ preference. Considering the relatively low costs of production for pharmaceuticals,^29,30^ the high sales incomes would mean supernormal profits for the originator companies, that is, returns on the factors of production (ie, resources needed to produce drugs) in excess of what would be necessary to maintain industry operation. As is well described, the pharmaceutical industry is one of the most profitable globally,^31,32^ and cancer drugs have been the largest source of their annual income, with their sales accounting for 11.7% of the global pharmaceutical market in 2017.^33^

The high incomes and supernormal returns would be less worrying if patients were able to access cancer drugs at affordable prices. However, the existing evidence suggests otherwise: access to cancer drugs globally remains low^34,35^ and the number of cancer drugs with annual costs at least in the tens of thousands is increasing fast.^19,36,37^ The resulting expenditure effects have compelled insurance schemes to exclude patients from coverage, restrict access, or impose high out-of-pocket costs.^34,38,39,40^ For these reasons, the high prices of cancer drugs have resulted in reduced capacity of health care systems to provide population-wide affordable access to cancer drugs.

Our analysis has also shown that the returns from cancer drugs are much higher than what would be considered a justifiable return required for rewarding and incentivizing innovation, both in economic terms and by reasonableness. Our results and observations from other published studies suggest that the excessive returns might have contributed to inefficiencies in R&D of cancer drugs and stifled clinically meaningful innovation. On inefficiencies in R&D of cancer drugs, leading experts in cancer research have observed considerable duplication of research effort (eg, checkpoint immune therapeutics).^41,42,43^ Companies seem to have adopted a derisking strategy by duplicating and pursuing marginal indications,^41,42,43^ with the expectation that the market would continue to bear the high prices irrespective of the magnitude of benefits in the name of innovation. A modeling study has shown that, hypothetically, a company might generate profit from running a clinical trials portfolio of chemically inert compounds by virtue of statistical probability.^44^ This is because of the high returns through high prices and the regulator’s willingness to grant approval based on a single randomized clinical trial with statistical significant results, even if the magnitude of benefit is marginal or is demonstrated through only surrogate end points.^44^ Over the longer term, such distortion of incentives and investment may stifle clinically meaningful innovation.

This lack of clinically meaningful innovation among cancer drugs is corroborated by extant evidence. A number of studies note that R&D in the past decades has resulted in many drugs with limited evidence to support their clinical value.^45,46,47,48,49^ For example, of the 54 cancer drugs approved by the FDA from 2008 to 2012, only one-third have evidence that they prolong survival, and only 5 additional drugs were shown to lead to improved overall survival during the postmarketing period.^48^ For drugs that improve survival, the overall magnitude of benefit was often small: FDA-approved drugs for solid tumors from 2002 to 2014 were found to result in modest progression-free and overall survival gains of 2.5 months and 2.1 months, respectively.^43^ In addition, there have been concerns about the safety profile of newer drugs. A meta-analysis of FDA-approved cancer drugs from 2000 to 2010 showed that the risk of toxic death and treatment discontinuation were greater for newer targeted drugs than the comparator products.^49^ In short, while there are a high number of blockbuster cancer drugs, there are relatively fewer drugs with clinically meaningful benefits, supporting the view that the majority of R&D of cancer drugs to date has not generated true innovation.

Furthermore, it could be argued that the returns from cancer drugs have been so high that they might have overincentivized the pharmaceutical industry to dedicate substantial, perhaps disproportionate level of investment toward the R&D of cancer drugs, possibly at the expense of research in other disease areas. Indeed, a review shows that there were 4006 randomized clinical trials for cancer drugs in 2017, representing about half of all pharmaceutical trials.^50^ This high level of investments suggests that companies must have deemed the highly favorable returns on investments for cancer drugs as more than sufficient to overcome their claims of higher investment risks due to higher R&D costs and failure and/or discontinuation rates of cancer drug trials. The considerable financial incentives might even have encouraged companies to take higher than normal risk in initiating expensive trials with low probability of success (eg, mitosis-targeting drug candidates^43^).

### Limitations

Our analysis has a number of limitations. First, it is limited by the availability and accuracy of data presented in the companies’ annual reports. These reports provide a comprehensive summary of a company’s financial performance as required legally, but as documents intended also to attract potential investors, they may report low-performing assets in aggregated format. Thus, our analysis might have omitted sales incomes from drugs with lower returns. However, our sensitivity analysis incorporated full R&D costs but 0 sales incomes for the 57 drugs excluded in the base-case showed that the overall income return per R&D dollar remained high ($8.80). For some drugs, (eg, belinostat) sales incomes might have been sufficient to cover R&D costs because of the significant public sector contribution toward its R&D. Second, owing to nonreporting of sales incomes for some products and in specific years, this study had to use mathematical derivation to estimate missing data. This is unlikely to affect the findings because only 7.6% of the data points were derived, and all were based on interpolation between 2 known values. Third, companies may conduct additional postapproval clinical trials for possible extension of indications or dosage forms. Our analysis showed that the overall findings were not sensitive to the costs of these trials. Finally, this study did not attempt to estimate cumulative profits (ie, sales incomes minus expenses and taxes). This is due to a lack of information around the magnitude and year-to-year variations in production costs specific to cancer drugs. Nonetheless, as noted above, profits from cancer drugs are likely to be supernormal, considering the low cost of production, long period of market exclusivity, and a net overall profit margin of 20% to 30% industry-wide.^31,51^

## Conclusions

This study has shown that cancer drugs, through their high prices, have generated substantial financial returns for the originator companies. Excessive returns on investment may distort R&D investment and stifle future clinically meaningful innovation, with evidence of such a trend having already emerged in the R&D of cancer drugs. High prices have restricted patient access to cancer drugs and compromised financial sustainability of health systems globally. For these reasons, actions must be performed to correct the unsustainable trend of drug prices, in cancer and other therapeutic areas.

## Notes

Supplement. **eTable.** Reported and Derived (shaded) Sales Incomes in US Dollar, by Molecule
