# Supplementary material for: Comparison of Sales Income and Research and Development Costs for FDA-Approved Cancer Drugs Sold by Originator Drug Companies
Source: JAMA Netw Open. 2019 Jan 4;2(1):e186875. doi: 10.1001/jamanetworkopen.2018.6875 (PMC6324319; doi:10.1001/jamanetworkopen.2018.6875)
Supplement: Supplement. — eTable. Reported and Derived (shaded) Sales Incomes in US Dollar, by Molecule [file jamanetwopen-2-e186875-s001.pdf]

Supplementary Online Content

Tay-Teo K, Ilbawi A, Hill SR. Comparison of sales income and research and development costs for FDA-approved cancer drugs sold by originator drug companies. *JAMA Netw Open*. 2019;2(1):e186875. doi:10.1001/jamanetworkopen.2018.6875

eTable. Reported and Derived (shaded) Sales Incomes in US Dollar, by Molecule

This supplementary material has been provided by the authors to give readers additional information about their work.

© 2019 Tay-Teo et al. *JAMA Network Open*.

eTable. Reported and derived (shaded) sales incomes in US dollar, by molecule

Nr	Medicine name	1989	1990	1991	1992	1993	1994	1995	1996	1997	1998	1999	2000	2001	2002	2003	2004	2005	2006	2007	2008	2009	2010	2011	2012	2013	2014	2015	2016	2017
**Medicines included**																														
1	Abiraterone																							301.0	961.0	1698.0	2237.0	2231.0	2260.0	2505.0
2	Acalabrutinib																													3.0
3	Aflibercept																								32.1	70.4	91.5	85.4	71.9	84.6
4	Alectinib																												184.8	367.5
5	Anastrozole								69.3	104.6	121.0	140.0	156.0	191.0	331.0	519.0	811.0	1181.0	1508.0	1730.0	1857.0	1921.0	1512.0	756.0	543.0	351.0	298.0	250.0	232.0	217.0
6	Aprepitant																		130.8	201.7	259.7	313.1	378.0	419.0	489.0	507.0	553.0	535.0	549.0	556.0
7	Atezolizumab																												159.4	494.4
8	Axitinib																								100.0	319.0	410.0	430.0	401.0	339.0
9	Azacitidine																				206.7	387.2	534.3	705.3	823.0	826.0	690.0	675.0	674.0	664.0
10	Belinostat																										5.0	10.1	13.4	12.4
11	Bendamustine																						9.1	40.1	43.9	40.0	34.9	33.9	41.4	64.2
12	Bevacizumab																555.1	1337.3	2362.0	3421.7	4807.9	5718.8	6194.6	5959.5	6145.0	7501.6	7005.5	6948.0	6886.3	6789.8
13	Bicalutamide								127.2	176.5	245.0	340.0	433.0	569.0	644.0	854.0	1012.0	1123.0	1206.0	1335.0	1258.0	844.0	579.0	550.0	454.0	376.0	320.0	267.0	247.0	215.0

© 2019 Tay-Teo et al. *JAMA Network Open*.

Nr	Medicine name	1989	1990	1991	1992	1993	1994	1995	1996	1997	1998	1999	2000	2001	2002	2003	2004	2005	2006	2007	2008	2009	2010	2011	2012	2013	2014	2015	2016	2017
14	Blinatumomab																											77.0	115.0	175.0
15	Bortezomib															59.6	143.1	192.1	220.5	265.2	1174.0	1426.7	1658.7	2002.0	2413.6	2634.4	3059.3	2671.4	2488.8	2338.1
16	Bosutinib																								15.0	29.2	57.0	111.0	167.0	233.0
17	Brentuximab																							7.5	56.4	139.3	216.1	228.0	276.7	343.2
18	Cabazitaxel																						108.6	261.5	302.1	306.8	362.1	355.9	396.0	435.2
19	Cabozantinib																										40.1	34.2	143.4	381.4
20	Capecitabine										20.7	59.9	88.8	154.0	284.8	382.3	429.6	639.4	774.3	959.2	1118.2	1399.8	1367.2	1524.8	1623.7	1627.8	847.2	533.3	513.7	459.9
21	Carfilzomib																								64.0	73.0	331.0	512.0	692.0	835.0
22	Ceritinib																										31.0	79.0	91.0	91.0
23	Cetuximab																336.8	684.1	1074.8	1335.0	1576.2	1651.1	1748.1	1880.2	1842.1	1867.6	1921.5	1497.3	1660.5	1607.6
24	Crizotinib																							16.0	123.0	282.0	438.0	488.0	561.0	594.0
25	Dabrafenib/Trametinib																									40.6	333.9	453.0	672.0	873.0
26	Daratumumab																											20.0	572.0	1242.0

© 2019 Tay-Teo et al. *JAMA Network Open*.

Nr	Medicine name	1989	1990	1991	1992	1993	1994	1995	1996	1997	1998	1999	2000	2001	2002	2003	2004	2005	2006	2007	2008	2009	2010	2011	2012	2013	2014	2015	2016	2017
27	Dasatinib																		25.0	158.0	310.0	421.0	576.0	803.0	1019.0	1280.0	1493.0	1620.0	1824.0	2005.0
28	Deferasirox																	2.0	143.0	357.0	531.0	652.0	762.0	850.0	870.0	893.0	926.0	917.0	956.0	1059.0
29	Degarelix																									7.2	23.6	28.1	35.8	40.1
30	Denosumab																						8.0	351.0	748.0	1019.0	1211.0	1405.0	1529.0	1575.0
31	Docetaxel							2.0	89.0	132.1	196.1	291.0	338.2	897.1	1186.3	1456.0	1783.9	2001.2	2198.2	2563.6	3187.4	2947.2	2810.6	1282.3	723.7	543.2	352.8	246.1	198.0	195.0
32	Durvalumab																													19.0
33	Elotuzumab																											3.0	150.0	231.0
34	Enzalutamide																								71.5	125.0	515.4	1133.5	2317.2	2247.6
35	Eribulin																						25.1	200.5	283.2	295.1	333.2	332.1	342.9	355.7
36	Erlotinib																96.6	310.8	648.3	885.0	1121.9	1198.5	1270.4	1715.8	1823.2	1819.5	1832.4	1631.6	1469.8	1169.7
37	Everolimus																					70.0	243.0	443.0	797.0	1309.0	1575.0	1607.0	1516.0	1525.0
38	Exemestane													47.0	52.2	58.0	143.0	247.0	320.0	401.0	465.0	483.0	483.0	361.0	210.0	185.0	122.0	86.6	61.5	43.7
39	Filgrastim			172.6	544.0	719.4	829.0	936.0	1016.3	1055.7	1116.6	1256.6	1223.7	1346.4	1379.6	1267.0	1175.0	1200.0	1213.0	1277.0	1341.0	1288.0	1286.0	1260.0	1260.0	1398.0	1159.0	1049.0	765.0	549.0

© 2019 Tay-Teo et al. *JAMA Network Open*.

Nr	Medicine name	1989	1990	1991	1992	1993	1994	1995	1996	1997	1998	1999	2000	2001	2002	2003	2004	2005	2006	2007	2008	2009	2010	2011	2012	2013	2014	2015	2016	2017
40	Fulvestrant														35.0	77.0	99.0	140.0	186.0	214.0	249.0	262.0	345.0	546.0	654.0	681.0	720.0	704.0	830.0	941.0
41	Gefitinib														67.0	228.0	389.0	273.0	237.0	238.0	265.0	297.0	393.0	554.0	611.0	647.0	623.0	543.0	513.0	528.0
42	Gemcitabine								113.0	174.8	306.8	455.8	559.3	722.9	875.0	1021.7	1214.4	1334.5	1408.1	1592.4	1719.8	1360.0	1149.4	452.1	156.3	102.5	46.5	15.6	12.7	10.3
43	Goserelin		81.2	114.1	160.2	225.0	316.0	408.5	528.0	577.0	626.0	686.0	734.0	728.0	794.0	869.0	917.0	1004.0	1008.0	1104.0	1138.0	1086.0	1115.0	1179.0	1093.0	996.0	924.0	816.0	816.0	735.0
44	Granisetron													224.5	289.3	324.4	367.7	401.6	397.1	354.2	105.5	91.9	62.7	66.7	41.2	36.1	29.0	23.3	18.7	15.0
45	Hpv Quadrivalent Vaccine																		234.8	1480.6	1402.8	1118.4	988.0	1209.0	1631.0	1831.0	1738.0	1908.0	2173.0	2308.0
46	Ibritumomab Tiuxetan														13.7	19.6	18.7	19.4	16.4	13.7	11.4	15.7	29.0	27.6	30.3	29.4	24.7	17.5	10.7	11.8
47	Ibrutinib																									13.6	748.0	1443.0	3083.0	4466.0
48	Idelalisib																										23.0	132.0	168.0	149.0
49	Imatinib													152.3	611.3	1128.0	1634.0	2170.0	2554.0	3050.0	3670.0	3944.0	4265.0	4659.0	4675.0	4693.0	4746.0	4658.0	3323.0	1943.0
50	Ipilimumab																							360.0	706.0	960.0	1308.0	1126.0	1053.0	1244.0
51	Irinotecan							14.8	59.0	154.0	194.0	293.0	441.0	613.0	574.0	299.0	554.0	910.0	903.0	969.0	563.0	332.0	117.0	105.5	95.2	85.9	77.4	69.8	63.0	58.0

© 2019 Tay-Teo et al. *JAMA Network Open*.

Nr	Medicine name	1989	1990	1991	1992	1993	1994	1995	1996	1997	1998	1999	2000	2001	2002	2003	2004	2005	2006	2007	2008	2009	2010	2011	2012	2013	2014	2015	2016	2017
52	Ixazomib																											33.9	270.2	413.7
53	Lapatinib																			102.0	187.5	263.2	350.9	370.2	377.6	323.4	281.3	242.1	209.0	180.4
54	Lenalidomide																	2.9	320.6	773.9	1324.7	1706.4	2469.2	3208.2	3767.0	4280.0	4980.0	5801.0	6974.0	8187.0
55	Lenvatinib																										3.8	95.0	197.6	287.1
56	Letrozole													197.8	271.0	296.9	386.0	536.0	719.0	937.0	1129.0	1266.0	1376.0	911.0	438.0	384.0	380.0	304.0	192.1	143.1
57	Leuprolide, Intramuscular											1349.3	1435.5	1483.6	1548.0	1562.1	1646.1	1617.3	1589.0	1698.0	1766.0	1846.0	2067.0	2322.4	2260.1	2084.2	1948.4	1853.7	1870.7	1848.9
58	Leuprolide, Subcutaneous																12.9	23.6	50.7	78.1	109.3	133.6	160.6	172.9	173.0	153.7	176.5	157.0	161.8	141.8
59	Nilotinib																				89.0	212.0	399.0	716.0	998.0	1266.0	1529.0	1632.0	1739.0	1841.0
60	Niraparib																													109.0
61	Nivolumab																										6.0	942.0	3774.0	4948.0
62	Obinutuzumab																											133.1	199.0	282.2
63	Octreotide										458.5	541.0	647.6	816.0	943.0	695.0	827.0	896.0	915.0	1027.0	1123.0	1155.0	1291.0	1443.0	1512.0	1589.0	1650.0	1630.0	1646.0	1612.0

© 2019 Tay-Teo et al. *JAMA Network Open*.

Nr	Medicine name	1989	1990	1991	1992	1993	1994	1995	1996	1997	1998	1999	2000	2001	2002	2003	2004	2005	2006	2007	2008	2009	2010	2011	2012	2013	2014	2015	2016	2017
64	Ofatumumab																					4.7	47.9	70.5	94.8	117.2	88.8	74.3	62.1	36.0
65	Olaparib																											94.0	218.0	297.0
66	Osimertinib																											19.0	423.0	955.0
67	Oxaliplatin											83.1	130.0	337.8	878.1	878.1	1515.5	1945.3	2124.2	2080.7	1401.2	1329.2	565.6	1489.6	1228.8	293.5	278.5	251.7	188.1	201.8
68	Paclitaxel					86.3	345.2	580.0	813.0	941.0	1206.0	1481.0	1592.0	1112.0	857.0	934.0	991.0	747.0	563.0	422.0	385.0	278.8	218.0	170.4	133.2	104.2	81.4	63.7	49.8	38.9
69	Paclitaxel Protein-Bound															18.0	62.0	133.7	174.9	324.7	335.6	314.5	351.6	385.9	427.0	649.0	848.0	967.0	973.0	992.0
70	Palbociclib																											723.0	2135.0	3126.0
71	Panitumumab																		39.0	170.0	153.0	233.0	288.0	322.0	359.0	389.0	505.0	549.0	611.0	642.0
72	Pazopanib																					1.6	58.7	160.3	289.1	517.2	674.3	565.0	729.0	808.0
73	Pegfilgrastim														464.0	1255.0	1740.0	2304.0	2710.0	3000.0	3318.0	3355.0	3558.0	3952.0	4092.0	4392.0	4596.0	4715.0	4648.0	4534.0
74	Pembrolizumab																										55.0	566.0	1402.0	3809.0
75	Pemetrexed																142.6	463.2	611.8	854.0	1154.7	1706.0	2208.6	2461.1	2594.3	2703.0	2792.0	2493.1	2283.3	2062.5

© 2019 Tay-Teo et al. *JAMA Network Open*.

Nr	Medicine name	1989	1990	1991	1992	1993	1994	1995	1996	1997	1998	1999	2000	2001	2002	2003	2004	2005	2006	2007	2008	2009	2010	2011	2012	2013	2014	2015	2016	2017
76	Pertuzumab																								59.7	351.7	1002.2	1502.1	1874.1	2229.4
77	Plerixafor																							82.1	123.4	134.1	147.2	158.5	168.1	183.8
78	Pomalidomide																								12.0	305.0	680.0	984.0	1311.0	1614.0
79	Ponatinib																												22.0	171.0
80	Pralatrexate																					50.5	35.2	38.0	41.1	44.4	45.0	45.6	46.2	43.0
81	Radium Ra 223 Dichloride																									55.0	208.2	284.9	366.2	460.0
82	Ramucirumab																										75.6	383.8	614.1	758.3
83	Regorafenib																								41.1	261.6	297.1	347.0	304.2	355.1
84	Ribociclib																													76.0
85	Rituximab										162.6	326.2	532.9	1004.1	1495.8	2060.1	2717.6	3336.5	3441.4	3736.1	4300.7	4552.9	4819.8	6039.4	5993.6	6213.6	6116.8	5872.1	6886.3	5920.8
86	Romidepsin																						15.8	30.9	50.0	54.0	66.0	69.0	80.0	76.0
87	Ruxolitinib																									163.0	279.0	410.0	581.0	777.0
88	Sorafenib																		163.1	369.4	676.4	838.9	933.8	1008.3	1018.0	1023.9	1025.2	988.9	962.4	940.2
89	Sunitinib																		219.0	581.0	847.0	964.0	1066.0	1187.0	1236.0	1204.0	1174.0	1120.0	1095.0	1081.0

© 2019 Tay-Teo et al. *JAMA Network Open*.

Nr	Medicine name	1989	1990	1991	1992	1993	1994	1995	1996	1997	1998	1999	2000	2001	2002	2003	2004	2005	2006	2007	2008	2009	2010	2011	2012	2013	2014	2015	2016	2017
90	Tamoxifen				265.0	304.0	348.7	400.0	450.8	508.0	526.0	573.0	576.0	630.0	480.0	178.0	134.0	114.0	89.0	83.0	85.0	88.0	89.0	99.0	48.7	40.9	34.3	28.8	24.1	20.2
91	Temozolomide											36.0	121.0	180.0	278.0	324.0	459.0	588.0	703.0	861.0	1002.0	1033.0	1065.0	935.0	917.0	708.0	350.0	312.0	283.0	271.0
92	Thalidomide										3.2	24.1	62.0	82.0	119.0	223.7	308.6	387.8	433.0	447.1	504.7	436.9	389.6	339.1	302.0	245.0	221.0	185.0	152.0	132.0
93	Topotecan													129.5	140.9	179.7	181.3	180.0	208.1	238.0	257.4	267.9	222.6	91.3	39.5	25.0	12.3	6.4	3.4	1.8
94	Trastuzumab										30.5	199.7	319.7	477.5	645.9	873.8	1154.5	1723.7	3131.6	4043.3	4701.8	4840.1	5205.2	5915.5	6278.3	6557.7	6850.4	6796.3	6885.3	7120.8
95	Trastuzumab Emtansine																									252.4	585.2	799.4	843.7	927.9
96	Trifluridine/Tipiracil																											77.7	276.7	290.6
97	Vemurafenib																							34.9	249.5	381.9	328.6	222.5	216.2	165.0
98	Vismodegib																								30.9	49.5	79.2	174.4	203.0	248.1
99	Zoledronic Acid														758.0	892.0	1078.0	1224.0	1283.0	1297.0	1382.0	1469.0	1511.0	1487.0	1288.0	605.4	558.9	418.7	313.7	235.0
Excluded drugs																														
100	Abarelix																													
101	Abemaciclib																													
102	Afatinib																													

© 2019 Tay-Teo et al. *JAMA Network Open*.

Nr	Medicine name	1989	1990	1991	1992	1993	1994	1995	1996	1997	1998	1999	2000	2001	2002	2003	2004	2005	2006	2007	2008	2009	2010	2011	2012	2013	2014	2015	2016	2017
103	Aldesleukin																													
104	Alemtuzumab																													
105	Alitretinoin																													
106	Arsenic Trioxide																													
107	Bexarotene																													
108	Brigatinib																													
109	Cladribine																													
110	Clofarabine																													
111	Cobimetinib																													
112	Copanlisib																													
113	Cytarabine																													
114	Daunorubicin																													
115	Decitabine																													
116	Denileukin Diftitox																													
117	Dinutuximab																													
118	Dronabinol Oral Solution																													
119	Enasidenib																													
120	Epirubicin																													
121	Fludarabine																													

© 2019 Tay-Teo et al. *JAMA Network Open*.

Nr	Medicine name	1989	1990	1991	1992	1993	1994	1995	1996	1997	1998	1999	2000	2001	2002	2003	2004	2005	2006	2007	2008	2009	2010	2011	2012	2013	2014	2015	2016	2017
122	Flutamide																													
123	Gemtuzumab Ozogamicin																													
124	Idarubicin																													
125	Imiquimob																													
126	Ixabepilone																													
127	Lantreotide																													
128	Lenograstim																													
129	Melphalan																													
130	Mitomycin																													
131	Nelarabine																													
132	Nilutamide																													
133	Olaratumab																													
134	Omacetaxine Mepesuccinate																													
135	Panobinostat																													
136	Pegaspargase																													
137	Porfimer																													
138	Rolapitant																													

© 2019 Tay-Teo et al. *JAMA Network Open*.

Nr	Medicine name	1989	1990	1991	1992	1993	1994	1995	1996	1997	1998	1999	2000	2001	2002	2003	2004	2005	2006	2007	2008	2009	2010	2011	2012	2013	2014	2015	2016	2017
139	Rucaparib																													
140	Sipuleucel - T																													
141	Sonidegib																													
142	Talimogene Laherparepvec																													
143	Temsirolimus																													
144	Teniposide																													
145	Thiotepa																													
146	Toremifene																													
147	Tositimomab																													
148	Tositumomab + Iodine																													
149	Trabectedin																													
150	Triptorelin																													
151	Uridine Triacetate																													
152	Valrubicin																													
153	Vandetanib																													
154	Venetoclax																													
155	Vinorelbine																													
156	Vorinostat																													

© 2019 Tay-Teo et al. *JAMA Network Open*.

© 2019 Tay-Teo et al. *JAMA Network Open*.
